# Self-Organization of Blood Pressure Regulation: Experimental Evidence

**DOI:** 10.3389/fphys.2016.00112

**Published:** 2016-03-31

**Authors:** Jacques-Olivier Fortrat, Thibaud Levrard, Sandrine Courcinous, Jacques Victor

**Affiliations:** CaDyWec Associated Lab, Faculté de Médecine d'Angers, UMR Centre National De La Recherche Scientifique 6214 Institut national de la santé et de la recherche médicale 1083 (Biologie Neurovasculaire et Mitochondriale Intégrée)Angers, France

**Keywords:** autonomic nervous system, baroreflex, Blood pressure control, heart rate variability, non-linear dynamics, self-organized criticality

## Abstract

Blood pressure regulation is a prime example of homeostatic regulation. However, some characteristics of the cardiovascular system better match a non-linear self-organized system than a homeostatic one. To determine whether blood pressure regulation is self-organized, we repeated the seminal demonstration of self-organized control of movement, but applied it to the cardiovascular system. We looked for two distinctive features peculiar to self-organization: non-equilibrium phase transitions and hysteresis in their occurrence when the system is challenged. We challenged the cardiovascular system by means of slow, 20-min Tilt-Up and Tilt-Down tilt table tests in random order. We continuously determined the phase between oscillations at the breathing frequency of Total Peripheral Resistances and Heart Rate Variability by means of cross-spectral analysis. We looked for a significant phase drift during these procedures, which signed a non-equilibrium phase transition. We determined at which head-up tilt angle it occurred. We checked that this angle was significantly different between Tilt-Up and Tilt-Down to demonstrate hysteresis. We observed a significant non-equilibrium phase transition in nine healthy volunteers out of 11 with significant hysteresis (48.1 ± 7.5° and 21.8 ± 3.9° during Tilt-Up and Tilt-Down, respectively, *p* < 0.05). Our study shows experimental evidence of self-organized short-term blood pressure regulation. It provides new insights into blood pressure regulation and its related disorders.

## Introduction

Short-term blood pressure control by means of the baroreflex is a prime example of a homeostatic model in which a biological variable is maintained at its normal value by means of a physiological regulatory mechanism (Kamiya et al., 2014). Blood pressure drifts, such as those caused by blood volume shifts arising with the standing position, are compensated heart beat after heart beat by the baroreflex (Rowell, 1993). Baroreflex inputs from blood vessel walls inform the central nervous system of these drifts. In return, the central nervous system alters heart and blood vessel functions to compensate for the drift and to return blood pressure to its reference value (Kamiya et al., 2014). The homeostatic model is based on causal relationships that draw a (curvi-) linear link between blood pressure and heart rate (Parlow et al., 1995).

This homeostatic model has limitations. The (curvi-) linear link is not spontaneously obvious and needs vaso-active drugs to be revealed (Parlow et al., 1995). A reference value (the set point) is central to a homeostatic model. A set point is supposed to be cast in stone into the central nervous system. However, it must constantly change to follow constant environmental changes: resetting. Integrating on its resetting into the homeostatic model is full of complexities (Koushanpour, 1991; Schwartz and Stewart, 2012). It is very difficult to imagine that a physiological system might integrate a panel of set point adapted to a broad range of environmental conditions. Lastly, the homeostatic model failed to explain the pathogenesis of the main diseases related to blood pressure dysregulation: hypertension and vasovagal syncope (Oparil et al., 2003; Raj, 2014; da Silva, 2014).

An alternative model to the homeostatic one might help to understand blood pressure regulation and related diseases. Actually, spontaneous dynamics of the cardiovascular system (beat after beat blood pressure and heart rate variability) exhibits non-linear patterns such as fractal scaling and asymmetry (Kobayashi and Musha, 1982; Klintworth et al., 2012). One of these alternative models could be self-organization as suggested by Struzik (2014). Self-organization is a broad trans-disciplinary concept and should not be confused with cardiovascular specific concept of auto-regulation (Müller and Osterreich, 2014). Self-organized systems are not centered on a main controller (the central nervous system in the case of the homeostatic baroreflex). Their apparently ordered dynamics results from interactions of the numerous controllers of the system and not from causal relationships between two (or few) of them.

**Figure 1:**
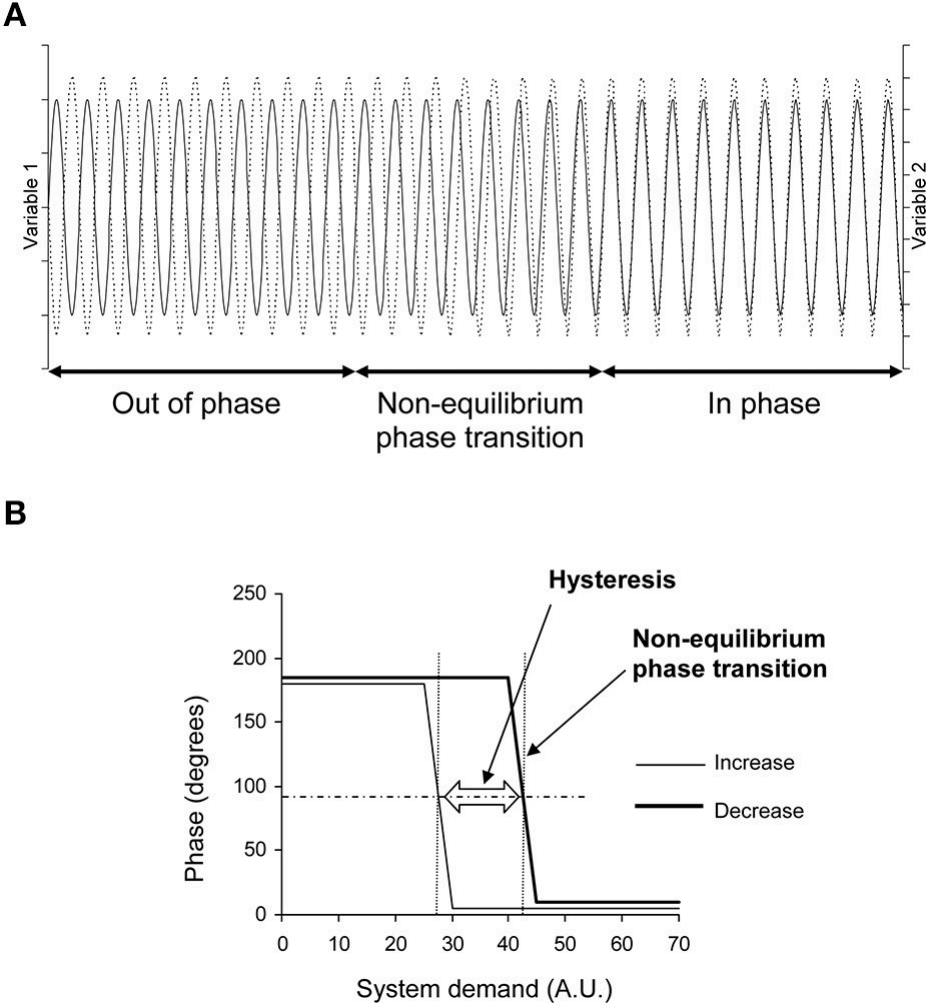
**Theoretical evolution of two variables related to two effectors of a self-organized system during demand increase and decrease on the system**. **(A)** Oscillations of these variables in the time domain (x axis) during demand increase on the system (for example). They are out of phase at the beginning (like a horse's front legs in a trot) and in phase at the end (as in a canter) with a transition between these two states: a non-equilibrium phase transition. This non-equilibrium phase transition is obvious in the time domain on this theoretical diagram because the two variables fluctuate with a single oscillation. Cardiovascular variable fluctuations are more difficult to observe because they are a mix of several oscillations. They require spectral analysis to be identified (see Figure 2). **(B)** Phase between these two variables during demand increase and decrease on the system. The phase shows two plateaus separated by a change during demand increase: a non-equilibrium phase transition. The phase shows the same pattern during demand decrease, but with a gap in comparison with demand increase: a hysteresis (AU, Arbitrary Unit). The horizontal dashed line indicates the middle between the two plateaus. Occurrence of the phase transition could be defined by the x-axis value of the intercept between the horizontal dashed line and the phase curve (vertical dotted line, one for each of the two phase curves). The difference between these occurrences quantifies the hysteresis between the two curves.

We hypothesized that the cardiovascular system is self-organized. To check this hypothesis, we repeated the seminal demonstration of self-organized control of movement, but applied it to the cardiovascular system (Schöner and Kelso, 1988; Kelso, 1995). This seminal demonstration experimentally showed two distinctive features peculiar to self-organization in the control of movement: occurrence of non-equilibrium phase transitions between two effectors of the regulatory system and hysteresis in their occurrence when system demand changes (Schöner and Kelso, 1988; Figure 1). Phase shifts between the evaluated variable (blood pressure) and one of the regulatory variables (related to an effector such as heart rate and peripheral resistance, etc.) may occur in homeostatic systems and have been observed (Saul et al., 1991). Phase shifts between two regulatory variables (or effectors) are not likely to occur in the case of homeostatic models while they are under the dependence of the same regulatory homeostatic system. Such a phase shift is then a non-equilibrium phase transition of a self-organized system (Schöner and Kelso, 1988; Kelso, 1995). Reverse hysteresis is commonly observed in physiological systems. However, these commonly observed hysteresis affect the variables themselves but not the phase between two regulatory variables that depend upon the same regulatory system. In this latter case, such a hysteresis is the signature of self-organization (Schöner and Kelso, 1988; Kelso, 1995).

In this study, we experimentally looked for phase transitions and hysteresis between two variables (heart rate and peripheral resistance) linked to two effectors of the cardiovascular system (heart and blood vessels, respectively) while we altered system demand by means of changes of position. We expected to observe such phase transitions and hysteresis in the case of a self-organized cardiovascular system, but not in the case of a homeostatic system.

## Materials and methods

### Goal of the procedure

This subheading provides an overview of the procedure. The methods are detailed in the following subheadings. The goal of our procedure is to demonstrate, in the cardiovascular system, first, the occurrence of non-equilibrium phase transitions, and seconds, hysteresis in their occurrence. Kelso (1995) illustrated these two features using horse gaits (a kind of movement control): the system observed is the motor system of the horse. Observations focus on two system effectors, whatever they may be; let us say the two front legs. Observations are performed while the system demand is increasing—in this case, running speed. The two front legs are out of phase at low speed (trot: a leg is stretched out, the other one, in). The horse shifts from trot to canter as speed increases and the two front legs are then almost in phase. This phase shift is a non-equilibrium phase transition. Such a phase shift is not supposed to occur in systems that are not self-organized. The phase between two wheels of a car remains unchanged when the car accelerates on a straight line. Moreover, the phase shift occurs at a different speed when slowing down (decreasing system demand) in the case of a self-organized system, meaning hysteresis of the phase shift—the second distinctive feature (Figure 1).

Our aim was to apply this experimental paradigm of the motor system to the blood pressure regulation system. We needed to select two cardiovascular variables from this system. Moreover, these variables had to be linked to two different effectors (two different “legs”), but not to blood pressure itself. We selected heart rate and Total Peripheral Resistance, which are linked to the heart and blood vessels, respectively. We increased system demand by means of a change in position (from supine to standing position). Standing is a cardiovascular challenge of particular significance. Vasovagal syncope is a very common transient dysfunction of blood pressure regulation that occurs mainly in the standing position (Raj, 2014; da Silva, 2014). Heart rate spontaneously oscillates at the breathing frequency (oscillation knows as sinus arrhythmia, Task Force of the European Society of Cardiology and the North American Society of Pacing and Electrophysiology, 1996). Sympathetic activity also spontaneously oscillates at this frequency and influences Peripheral Resistance (Häbler et al., 1994; Malpas, 1998; Briant et al., 2015). We monitored the phase between heart rate and Total Peripheral Resistance oscillation at the breathing frequency during change in position in order to look for a shift in this phase during the procedure. We repeated this procedure, but during a change in position in the opposite direction (from standing to supine) in order to decrease system demand. This repetition was to check whether the phase shift would occur with another delay during the posture change, indicating the occurrence of hysteresis in the phase shift.

### Ethical approval

Subjects received a complete description of the experimental procedures before giving their written informed consent. The Comité de Protection des Personnes d'Angers, France, approved the experiment which was in accordance with the Declaration of Helsinki, Finland.

### Subjects

Twelve healthy subjects participated in this study (mean ± SEM, age: 27.1 ± 1.8 years, weight: 62.1 ± 1.6 kg; height: 1.71 ± 0.01 m, 3 female). Based on their interview, they had no known cardiovascular disease. They had a normal medical exam including breathing frequency (from 10 to 19 breaths/min), supine and standing blood pressure, and electrocardiogram.

### Procedure

We monitored their blood pressure, electrocardiogram, and thoracic circumference (Finometer, FMS system, Amsterdam, Netherlands, a belt maintained the cuff at the heart level; ECG100C and respiratory belt TSD201 with a high pass filter set at 0.05 Hz and the low pass set at 1 Hz, MP150, Biopac system, Varra, Bulgaria) during two slow changes of position (20 min) obtained by means of a motorized tilt table (Akron A8622, Electro-Medical Equipment, Marietta, GA, USA). One change of position was from supine to head-up position (inclination of the tilt table from 0 to 70°, Tilt-Up) and the other one was from head-up position to supine (70 to 0°, Tilt-Down). We randomized the order of the changes of position, which were separated by a 7-day interval. We did not perform the second position change immediately after the first because of the duration of such a procedure, which could excessively challenge the cardiovascular system and increase the risk of a vasovagal reaction during the procedure (Natale et al., 1995). Monitoring began 5 min before the start of the change of position and extended until 5 min after its end (5 min in the initial position, 20 min of position change, and 5 min in the final position). Subjects were equipped with the monitoring material in the initial position of the procedure during a 10 min period in order to allow for adaptation to this position. The slow changes of position in our procedure, taking 20 min from supine to head-up position, did not match the faster everyday life action of standing up. We took into account dynamics of the fluid shift during standing, the delay of vasovagal syncope occurrence after standing, the period of spontaneous oscillations of cardiovascular variables, and the time resolution of our analysis tools (Fitzpatrick et al., 1991; Rowell, 1993; Task Force of the European Society of Cardiology and the North American Society of Pacing and Electrophysiology, 1996; Custaud et al., 2002). Lastly, a slow procedure should provide progressive phase transitions that are very different from the false, sudden phase transitions linked to spectral wrapping and inherent to spectral analysis.

### Analysis

**Figure 2:**
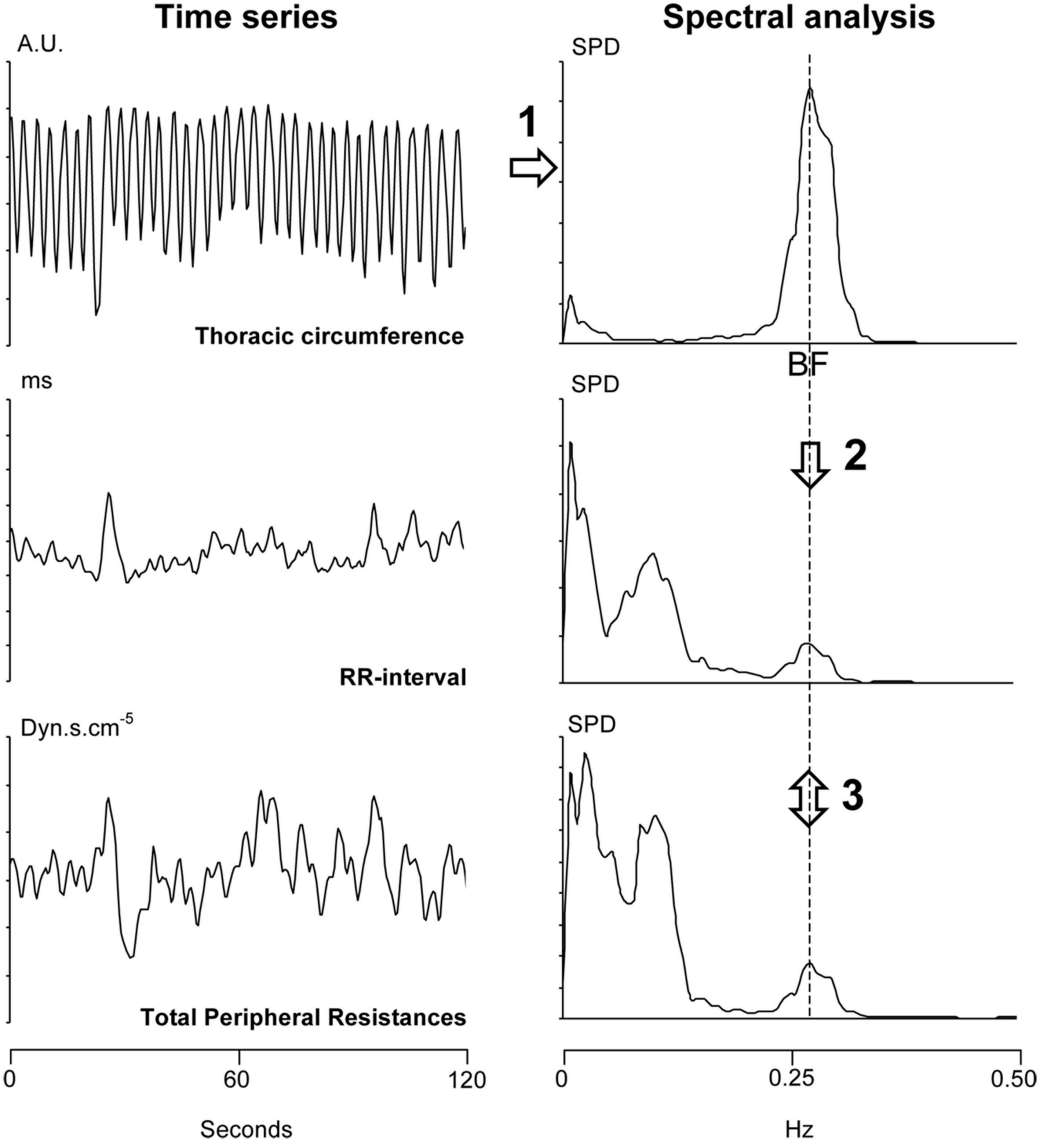
**Summary of the analysis of a 2-min long windows (overlapping each 30 s) from a 30-min long signal recording**. Step 1: We determined the breathing frequency (BF) by means of spectral analysis (upper right panel) of the thoracic circumference time series (in arbitrary units, AU, upper left panel). Step 2: We reported the BF on the spectral analysis of the RR-interval and Total Peripheral Resistances (two lower right panels). Step 3: We determined the phase and the coherence between the RR-interval and Total Peripheral Resistances at this BF (cross spectral analysis). SPD: spectral power density.

The 30-min long signals obtained from monitoring (blood pressure, electrocardiogram, and thoracic circumference) were stored on a computer for later analysis (MP150, Biopack system, Varra, Bulgaria, the sampling frequency was set at 1000 Hz, the accuracy of this system is 12 bits). We determined beat-by-beat RR-interval (the delay between two heart beats, the inverse of heart rate) from the electrocardiogram signal. We determined Total Peripheral Resistances from the blood pressure signal by means of the Model flow method (Bogert and van Lieshout, 2005). We determined thoracic circumference at each heartbeat. We re-sampled these three time series at 2 Hz. Figure 2 provides a summary of the mathematical analysis of these time series. We analyzed these time series by means of short-term fast Fourier transform with a moving window of 2 min overlapping each 30 s (Custaud et al., 2002; TSAS freeware, Yamamoto Y, University of Tokyo, Tokyo). The cardiovascular system spontaneously oscillates at two main frequencies (Mayer's wave and respiratory sinus arrhythmia, around 0.1 Hz and 0.25 Hz in humans, respectively; Task Force of the European Society of Cardiology and the North American Society of Pacing and Electrophysiology, 1996; Billman, 2011). We focused on respiratory sinus arrhythmia. We determined the breathing frequency for each of the windows as the maximum spectral power of the high frequency domain (from 0.15 to 0.40 Hz; Task Force of the European Society of Cardiology and the North American Society of Pacing and Electrophysiology, 1996) on the thoracic circumference time series (Figure 2, upper right panel). We performed a cross spectral analysis between the RR-interval and Total Peripheral Resistances time series (TSAS freeware, Yamamoto Y, University of Tokyo, Tokyo). For each of the windows, we determined the phase and the coherence between oscillations of the RR-interval and Total Peripheral Resistances arising from respiration at this breathing frequency (cross spectral analysis of RR-interval vs. Total Peripheral Resistances). We checked whether the determined phase was correctly associated with the same multiple of 360° as the preceding determined one. We were able to draw a graph by plotting this phase (y-axis) against the time (x-axis). We transformed the time scale of the x-axis (from 0 to 30 min) into the angle of the tilt table (from 0 to 70°).

For each subject, we checked whether the mean phase ± 2 standard deviations during the 5 min in the initial and final positions during this procedure did not overlap to demonstrate a non-equilibrium phase transition (see Bardy et al., 2002). We defined the occurrence of the phase transition as the x-axis value (in head-up tilt angle, see Figure 1B) of the measurement step for which the y-axis value is the closest to the mean phase between the initial and final positions (y axis of the phase transition plot, see Figure 1B) in cases with non-equilibrium phase transition. We compared this occurrence during Tilt-Up and Tilt-Down to check for hysteresis.

### Statistics

We performed comparisons of RR-interval, blood pressure, Total Peripheral Resistances, and breathing frequency between the horizontal supine and head-up position and between the Tilt-Up and Tilt-Down procedures by means of a Friedman's test. We applied a *post-hoc* Wilcoxon's test when appropriate (SPSS 9.0, Chicago, IL, USA). We performed a comparison of phase and hysteresis by means of circular statistics (test on the angular deviation; Mello, 2005). We set statistical significance at *p* ≤ 0.05.

## Results

**Figure 3:**
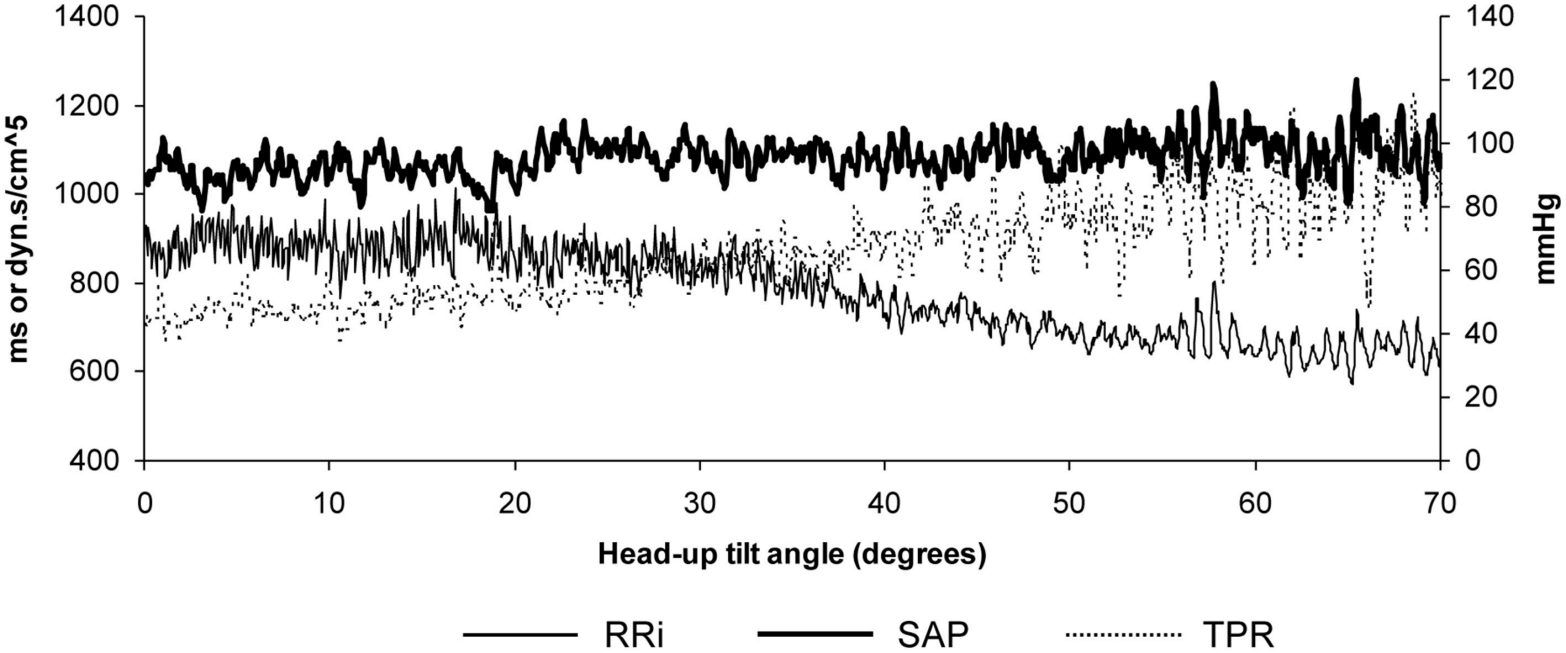
**Example of data recorded in a subject during a slow change of position from supine to the 70° head-up position in 20 min**. Note that Systolic Arterial Pressure (SAP) is maintained well-during the procedure thanks to the progressive decrease of the RR-interval (RRi; increase in heart rate) and to the progressive increase of the Total Peripheral Resistances (TPR).

**Figure 4:**
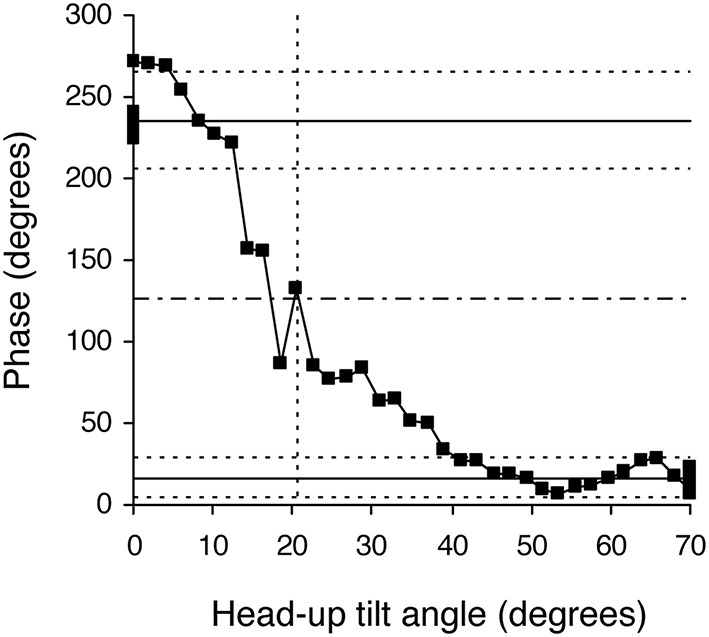
**Example of a phase diagram obtained in a healthy subject during a slow change of position from the 70° head-up position to supine**. Black squares indicate each measurement step on the 30-min long signals. This diagram of the phase relationship between oscillations of RR-interval and of Total Peripheral Resistances at the breathing frequency shows a non-equilibrium phase transition. Note that the whole phase transition occurred slowly, over more than 20 steps, a picture that is very different from sudden spectral wrapping. This diagram also illustrates the methods used to confirm that a phase transition occurred and to define its occurrence time (in head-up tilt angle units). The solid horizontal lines indicate the mean phase during the 5 min in the initial and final positions. The dotted line above and below each solid line indicates plus and minus two standard deviations of these mean phases, respectively. The upper and lower areas defined by the horizontal dotted lines do not overlap, confirming the phase transition. The horizontal dashed line indicates the middle between the two solid lines and identifies the measurement step with the closest y-value. We defined the occurrence of the phase transition as the x-axis value of this measurement step (in head-up tilt angle units, x axis, vertical dotted line; see Figure 1).

**Figure 5:**
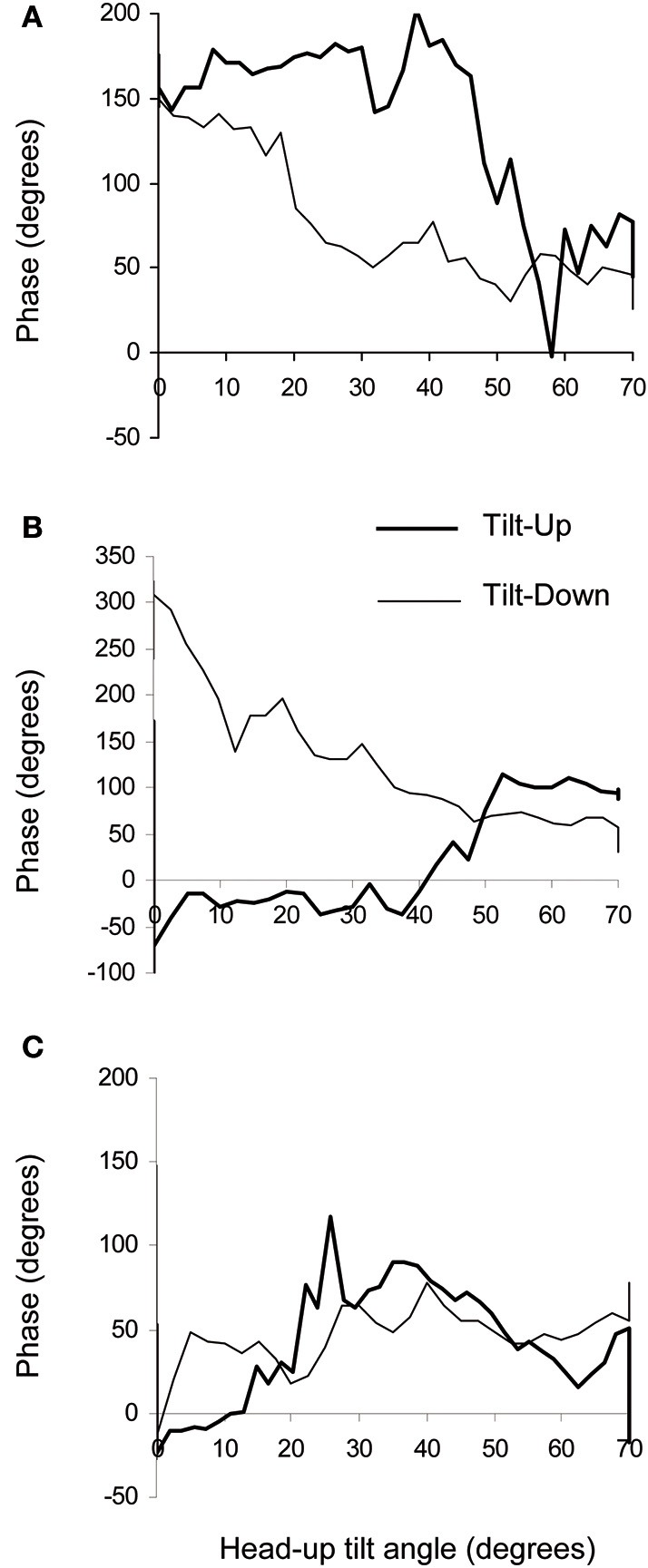
**Examples of phase diagrams obtained in healthy subjects during slow changes of position from supine to the 70° head-up position (Tilt-Up) and from the 70° head-up position to supine (Tilt-Down)**. These diagrams show the phase relationship between oscillations of the RR-interval and of the Total Peripheral Resistances at the breathing frequency. **(A)** Example of a diagram showing a non-equilibrium phase transition during both changes of position with a hysteresis between Tilt-Up and Tilt-Down (6 subjects out of 11). This graph resembles the theoretical graph shown in Figure 1B. **(B)** Example of a diagram showing a non-equilibrium phase transition during Tilt-Down but not during Tilt-Up (2 subjects out of 11). **(C)** Example of a diagram showing no phase transition during both Tilt-Up and Tilt-Down (2 subjects out of 11). The maximum y values are not the same on the different panels.

**Figure 6:**
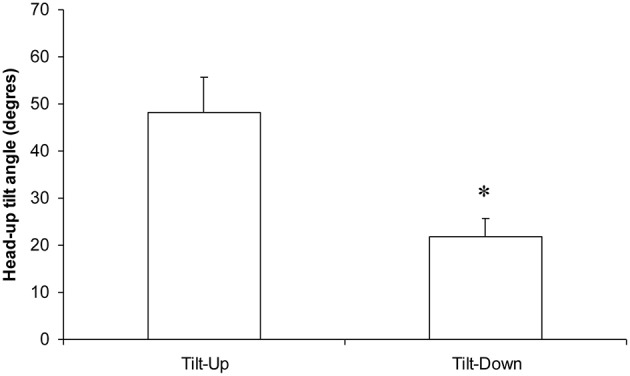
**Occurrence (in the head-up tilt angle, see the x axis of the phase transition plot in Figure 4) of the phase transition during slow changes of position from supine to the 70° head-up position (Tilt-Up) and from the 70° head-up position to supine (Tilt-Down)**. The significant difference indicates the hysteresis (^*^p < 0.05, mean ± SEM, *N* = 9).

The duration of the Tilt-Up procedure was 21′23″ ± 1′12″ and that of the Tilt-Down procedure was 20′47″ ± 1′10″. We excluded a subject from the analysis because of occurrence of ectopic beats during monitoring. Figure 3 shows the RR-interval and Total Peripheral Resistance time series of one of the subjects during Tilt-Up. RR-interval variability (or Heart Rate Variability) is obvious in this Figure, as is Total Peripheral Resistances variability. Figure 2 also shows this variability in the time domain (two lower left panels) and in the frequency domain (two lower right panels). Figure 3 also shows systolic blood pressure during the procedure. This subject maintained his systolic blood pressure well during the position change thanks to a decrease in the RR-interval (increase in heart rate) and an increase in Total Peripheral Resistances as usually described for cardiovascular adaptation to the head-up position (parasympathetic withdrawal and sympathetic activation; Rowell, 1993). Table 1 shows that the whole group followed such an adaptation to head-up position. Diastolic blood pressure increase is also expected during a change of position from supine to 70° head-up (Table 1; Rowell, 1993). This increase reflects overall vasoconstriction (Rowell, 1993). Breathing frequency did not change during the procedures (Table 1). Table 2 shows the changes of the phase between respiratory oscillations of the RR-intervals and Total Peripheral Resistances during position changes. Coherence between these two variables was good at the breathing frequency (0.58 ± 0.06 and 0.68 ± 0.03 during Tilt-Up and Tilt-Down, respectively). Nine subjects (out of 11) presented non-equilibrium phase transitions (Figure 4). Of these nine subjects, only six presented the typical hysteresis pattern of self-organized criticality with a non-equilibrium phase transition during both Tilt-Up and Tilt-Down (Figure 5A). The three remaining subjects among these nine presented a non-equilibrium phase transition during only one procedure out of the two indicating an extreme hysteresis that occurred outside the x-scale in one of the procedures (Figure 5B). Lastly, only two subjects did not show any phase behavior suggesting self-organization (Figure 5C). Statistical analysis of the nine subjects with non-equilibrium phase transitions identified a significant hysteresis between Tilt-Up and Tilt-Down, as demonstrated by the occurrence of phase transition at a significantly different head-up tilt angle depending upon whether in the Tilt-Up or Tilt-Down procedure (Figure 6). Moreover, when we look at the whole group of subjects, the phase between the respiratory oscillations of the RR-intervals and Total Peripheral Resistances in the supine position was slightly but significantly different between the Tilt-Up and Tilt-Down procedures, also indicating hysteresis (Table 2).

**Table 1:** **Cardiovascular and respiratory variables**.

	**Tilt-Up**		**Tilt-Down**	
	**Supine**	**70° Head-up**	**Supine**	**70° Head-up**
HR (bpm)	68.6±3.4	87.3±4.2	67.0±3.2	90.6±5.3
TPR (Dyn.s.cm^−5^)	727±47	1050±92	777±50^*^	1102±110
SAP (mmHg)	113.5±5.2	123.2±8.3	112.8±2.5	118.3±5.0
DAP (mmHg)	55.2±4.4	72.9±6.5	57.6±2.9	73.8±4.4
BF (Hz)	0.25±0.08	0.28±0.09	0.29±0.09	0.27±0.09

Mean ± SEM of the main cardiovascular and respiratory variables (HR, heart rate in beat per minute; TPR, Total Peripheral Resistances; SAP, Systolic Arterial Pressure; DAP, Diastolic Arterial Pressure; BF, Breathing Frequency) during slow changes of position on a tilt table. Changes of position were from supine to 70° head-up (Tilt-Up) or from 70° head-up to supine (Tilt-Down). HR, TPR, and DAP, but not SAP and BF, are significantly different between Supine and 70° Head-up, as expected (p < 0.000).

* p ≤ 0.05 vs. Supine during Tilt-Up procedure (Friedman).

**Table 2:** **Phase shift during the procedure**.

	**Tilt-Up**		**Tilt-Down**	
	**Supine**	**70° Head-up**	**Supine**	**70° Head-up**
Phase (degree)	107 ± 37	24 ± 37	223 ± 33^*^	65 ± 16^**^

*Phase shift (Mean ± SEM) between oscillations of RR-intervals and of Total Peripheral Resistances at the breathing frequency during slow changes of position on a tilt table. Changes of position were from supine to 70° head-up (Tilt-Up) or from 70° head-up to supine (Tilt-Down)*.

* p ≤ 0.05 vs. Supine during Tilt-Up procedure and

** *p ≤ 0.01 vs. Supine during Tilt-Down (test on the angular deviation)*.

## Discussion

We have demonstrated distinctive features peculiar to self-organization in the healthy cardiovascular system. We have also shown that the challenged cardiovascular system behaves like a self-organized system. Our study challenges the classical homeostatic view of short-term blood pressure regulation. Our experimental demonstration of a self-organized cardiovascular system is in accordance with the general theory of non-equilibrium in natural systems (Bak, 1996). It also supports the idea suggested by Mora and Bialek (2011) that biological systems are self-organized.

### Self-organization of blood pressure regulation: mathematical evidence

Our study provides experimental evidence of a self-organized cardiovascular system, but mathematical evidence has been observed in cardiovascular time series. The 1/f pattern of cardiovascular time series has been well-known since its description in heart rate variability (Kobayashi and Musha, 1982) and is typical of the dynamics of self-organized systems (Bak et al., 1987). Kalda et al. (2001) also showed Zipf's law in heart rate time series. Kiyono et al. (2005) observed phase transitions in heart rate variability during sleep and strenuous exercise.

### Self-organization in biological systems

Self-organization was first described in systems made of many interacting subunits. Self-organization accurately describes behavior in social animals despite the lack of a leader, such as a flock of birds, an ant hill, or a swarm of bees (each individual is a subunit; the queen's role is only concerns reproduction and not organization in the last two cases; Camazine et al., 2001). It is difficult to imagine how the behavior of a flock of birds could apply to a physiological function and to the cardiovascular system of a single organism. However, Schöner and Kelso (1988) and (Haken et al., 1985) demonstrated that a chain of muscles needs self-organization to produce a coordinated movement. Such a movement implies either muscle contractions or relaxations depending upon the phase of the movement (and some muscles must relax when others contract). It also involves cortical processes like decision making and learning, associated with multiple sense perceptions (eyesight, touch, and also proprioception and inner ear), and the stretch reflex. Central integration of these processes and continuous feedbacks would lead to cumulative delays that are not in accordance with the fast and accurate movements needed to cope with everyday activities and survival (Kelso, 1995). Self-organization provides an efficient way to coordinate these different kinds of processes (Kelso, 1995). An unconsciously regulated physiological function, balance control, is also self-organized (Bardy et al., 2002). However, this function also involves chains of muscles, multiple sense perceptions, and cortical processes (deciding to hold the banister). Blood pressure control is also unconscious, but it is not supposed to include cortical processes and sense perceptions. But obtaining a blood pressure adapted to various environmental conditions requires taking various inputs into account, both internal and external. Beat-by-beat blood pressure control involves not only the baroreflex but also low-pressure cardiopulmonary sensitivity, the renin-angiotensin-aldosterone-system, and many other systemic, regional, and local regulations (Guyton et al., 1972 gave a picture of the complexity of blood pressure control). Moreover, fast and accurate blood pressure control is also critical for survival, at least for the blood pressure response to fight or flight reactions. Self-organization could be an efficient way of coordinating all these regulatory mechanisms.

### Self-organization: a better understanding of blood pressure regulation

Self-organization provides a better understanding of blood pressure regulation. Self-organization explains some characteristics of the cardiovascular system that do not match with the homeostatic model: flexibility and robustness. Flexibility is best illustrated by mouth functions. The mouth is for eating, but one can also speak, whistle, play trumpet, kiss, etc., with this single organ (Kelso, 1995). Flexibility also refers to the adaptation of the system to numerous environments. This adaptation can be almost instantaneous, even when the organism encounters a new environment it has never encountered before or even during the human evolution. The cardiovascular system adapts well to high and low environmental temperatures, high altitude, diving, and even space flights. Non-linear self-organized systems are flexible, but homeostatic ones are not. Robustness refers to the lack of effect of a subunit failure on the whole system dynamics. Removing a single fish from a shoal will not affect the whole shoal's behavior. Carotid endarterectomy is a common surgical procedure to remove carotid artery stenosis that also removes baroreceptors, while blood pressure is only temporarily affected (Eliasziw et al., 1998). Patients can stand as well as before the surgical procedure. Non-linear self-organized systems are robust but homeostatic ones are not. Moreover, the spontaneous catastrophes that occur in self-organized systems may explain the occasional and transient failure of blood pressure regulation commonly seen in healthy subjects and known as vasovagal syncope. A better understanding of blood pressure regulation should also provide a better view of the pathogenesis of hypertension.

### Other arguments for self-organized blood pressure regulation

The study of self-organization of short-term blood pressure control is very new and other experimental studies on this topic are needed for an in-depth discussion. However, some authors have reported results consistent with a self-organized cardiovascular system. Several authors have described positive feedback mechanisms involved in blood pressure adaptation to environmental changes (Pagani et al., 1982; Legramante et al., 2001) Self-organization is the result of interactions between regular negative feedback mechanisms (as in the case of homeostatic physiological regulatory models including the baroreflex) and positive feedback mechanisms (Camazine et al., 2001). The design of some studies includes progressive position change (actual or simulated). These studies report a delayed progressive increase in heart rate that remains unchanged at the start of the procedure (until about 20° of head-up tilt angle), while other variables begin progressively to increase or decrease right from the start of the procedure (Johnson et al., 1974; Bahjaoui-Bouhaddi et al., 2000). These unparallel changes of two cardiovascular variables may reflect non-equilibrium phase transitions. Only a few studies have dealt with reverse position change (from standing to supine). Tomaselli et al. (1987) observed hysteresis in response to descending and ascending lower body negative pressure. These authors did not, however, look for phase relationships between variables. The hysteresis they reported is not the one that is a distinctive feature of self-organized systems, but rather the one commonly seen in many physiological variables. Lastly, we provide in a parallel publication (Fortrat and Gharib, 2016) a clinical evidence of self-organized blood pressure regulation by demonstrating a Guntenberg-Richter law of vasovagal reaction occurrence.

### Study limitation

Model-flow provides reliable measurements for a beat-by-beat analysis (Bogert and van Lieshout, 2005). However, it has been validated for applications different from the estimation of respiratory fluctuations in Total Peripheral Resistances. We focused on these fluctuations for two main reasons. First, cardiovascular fluctuations at the breathing frequency result from forcing that is external to the cardiovascular system (respiratory movements) like the experimental oscillations induced in the seminal demonstration that inspired our study (Haken et al., 1985; Schöner and Kelso, 1988; Kelso, 1995; Bardy et al., 2002). On the other hand, Mayer's wave is intrinsic within cardiovascular system (Julien, 2006). Its phase transitions, if any, would not necessarily sign self-organization. Second, analysis of high frequency respiratory fluctuations is more reliable than that of low frequency Mayer's wave when using a short-term fast Fourier transform (Task Force of the European Society of Cardiology and the North American Society of Pacing and Electrophysiology, 1996). Estimation of the respiratory fluctuations of Total Peripheral Resistances by means of Model-flow has limitations, but it enabled us to perform this non-invasive study on healthy volunteers. Invasive cardiovascular studies are conceivable on animal models, but they are not appropriate for the study of standing (see Rowell, 1993). Moreover, it is unlikely that an artifact behaves like a self-organized system rather than like a random process.

### Conclusion

Our study demonstrated self-organization of cardiovascular regulation. In other words, our study demonstrates that rather than baroreflex homeostatic blood pressure control, the many interactions of all the physiological mechanisms influencing the cardiovascular system give a sense of blood pressure control. This conclusion opens new fields of research into the understanding of blood pressure control and related disorders such as hypertension. Critical slowing-down, that is a prolonged recovery delay after a perturbation, is another distinctive feature peculiar to self-organized systems. Such a feature may also help to understand acute cardiovascular dysfunction such as life-threatening cardiac arrhythmias.

## Author contributions

JOF: conception, design, and drafting of the work; JOF, SC, TL: acquisition, analysis; JOF, SC, TL, JV: interpretation of data, critical revision of the work for important intellectual content and final approval of the version to be published.

## Funding

This study was supported by the Centre National d'Etudes Spatiales (CNES, grant # 2014/4800000763), and the Région Pays de la Loire. The funders had no role in study design, data collection and analysis, decision to publish, or preparation of the manuscript.

### Conflict of interest statement

The authors declare that the research was conducted in the absence of any commercial or financial relationships that could be construed as a potential conflict of interest.
